# Surgical Residents’ Perception of Feedback on Their Education: Protocol for a Scoping Review

**DOI:** 10.2196/56727

**Published:** 2024-08-19

**Authors:** Carlos Dario da Silva Costa, Gabriela Gouvea Silva, Emerson Roberto dos Santos, Ana Maria Rita Pedroso Vilela Torres de Carvalho Engel, Ana Caroline dos Santos Costa, Taisa Morete da Silva, Washington Henrique da Conceição, Helena Cristóvão, Alba Regina de Abreu Lima, Vânia MS Brienze, Thaís Santana Gastardelo Bizotto, Antonio Hélio Oliani, Júlio César André

**Affiliations:** 1 Center for Studies and Development of Health Education Faculty of Medicine of São José do Rio Preto São José do Rio Preto, São Paulo Brazil; 2 University Hospital Center Cova da Beira University of Beira Interior Covilhã Portugal

**Keywords:** medical education, occupational training, surgical residents, feedback, perception

## Abstract

**Background:**

Feedback is an essential tool for learning and improving performance in any sphere of education, including training of resident physicians. The learner’s perception of the feedback they receive is extremely relevant to their learning progress, which must aim at providing qualified care for patients. Studies pertinent to the matter differ substantially with respect to methodology, population, context, and objective, which makes it even more difficult to achieve a clear understanding of the topic. A scoping review on this theme will unequivocally enhance and organize what is already known.

**Objective:**

The aim of this study is to identify and map out data from studies that report surgical residents’ perception of the feedback received during their education.

**Methods:**

The review will consider studies on the feedback perception of resident physicians of any surgical specialty and age group, attending any year of residency, regardless of the type of feedback given and the way the perceptions were measured. Primary studies published in English, Spanish, and Portuguese since 2017 will be considered. The search will be carried out in 6 databases and reference lists will also be searched for additional studies. Duplicates will be removed, and 2 independent reviewers will screen the selected studies’ titles, abstracts, and full texts. Data extraction will be performed through a tool developed by the researchers. Descriptive statistics and qualitative analysis (content analysis) will be used to analyze the data. A summary of the results will be presented in the form of diagrams, narratives, and tables.

**Results:**

The findings of this scoping review were submitted to an indexed journal in July 2024, currently awaiting reviewer approval. The search was executed on March 15, 2024, and resulted in 588 articles. After the exclusion of the duplicate articles and those that did not meet the eligibility criteria as well as the inclusion of articles through a manual search, 13 articles were included in the review.

**Conclusions:**

Conducting a scoping review is the best way to map what is known about a subject. By focusing on the feedback perception more than the feedback itself, the results of this study will surely contribute to gaining a deeper understanding of how to proceed to enhance internal feedback and surgical residents’ learning progress.

**Trial Registration:**

Open Science Framework yexb; https://osf.io/yexkb.

**International Registered Report Identifier (IRRID):**

PRR1-10.2196/56727

## Introduction

### Background

Feedback is the delivery of information based on direct observation [1] that must always generate an action in the learner [2]. Feedback constitutes specific information about the comparison between learners’ performance or knowledge regarding a task and the desired standard, with the objective of learners seeking to improve their performance and reduce the distance between the ideal and the real [3]. In other words, feedback is a process in which learners acquire knowledge about their performance and use it to improve the quality of their work or learning strategies [4].

Thus, feedback is an essential tool for learning and improving performance in any sphere of education, especially in medical education and surgery [5-8]. Although the mechanism is not fully known, feedback represents an important part of the resident physician’s training [9], which focuses on the goal of providing qualified care for patients [1,10]. For this reason, the delivery of feedback to residents continues to be an important area of study in medical teaching [5,11].

There are different ways of giving feedback to learners: orally, in writing, through simulation, by videotaping learners, using audience response systems, and via computer-based and patient feedback tools, among others [6,11-13]. Likewise, many methodologies concerning the delivery of feedback have been described, including a feedback sandwich, Pendleton, Pendleton Plus, learning conversation, in groups, among peers, multisource feedback, and self-feedback [7,14-16]. However, independent of the approach, all modes of feedback must focus on the following 3 learning domains: cognitive, psychomotor, and affective [17].

The large number of methodologies in this field reflects the former paradigm related to feedback, where the key research question was “How can we develop the best feedback?” Accordingly, studies in this field traditionally focused on the quality of the information to be transmitted, how to transmit such information, and what would be the best moment to transmit the information [10]. However, the paradigm has since changed, with the crucial question to answer shifting to “How do learners become attentive to feedback and use the information they receive more effectively?” [18]. Thus, the priority has become the progress achieved in learning and the learner’s engagement in the process enabled by the feedback, which is encompassed by the term “feedforward” [16]. Recent studies on the subject have largely focused on this path [2,7,10,15,19].

Therefore, how the feedback is perceived and the reaction it generates in the learner is as important (or even more important) as the methodology that is used, the moment when it is given, or the information that is provided. Studies have shown that medical students and residents consider that the amount of feedback they receive is insufficient [11,12,20]. Furthermore, they often have a different perception of the feedback received compared to that of the person delivering the feedback [3,9,18,19], although some studies have shown that residents and teachers can share similar opinions concerning the qualities of effective feedback [10]. Feedback is considered effective when it can generate results and promote positive and desirable development [3]. Some factors that reportedly influence the perception of feedback include the teacher’s credibility in the opinion of the learner, who is responsible for giving feedback when the teams are multidisciplinary, the manner and the environment in which feedback is given, the teacher’s educational beliefs, the learner’s individual experiences, the learner’s level of expectation and motivation, and the relationship between feedback and the reflection it generates in the learner [5,7,11,15,21,22].

In opposition to active (or external) feedback, in which the teacher has a primordial role in the learning process, the reflection that the feedback generates in the learner (or internal feedback) enables the learner to go through stages of the competence awareness theory [23], developing a metacognitive learning process, a step that is inseparable from the intentional learning process [3]. That is, the learner’s perception of the feedback they receive is extremely relevant to their learning progress. Moreover, the incorporation of metacognitive aspects in the learning process equips the student with skills to become a lifelong learner [24].

### Prior Work

Surgical residents recognize that feedback provides useful suggestions for future improvement, and the lack of feedback can cause frustration [25] or affect self-confidence [26]. They prefer to receive feedback during or immediately after a case, in a face-to-face manner, and value the feedback much more when received within 1 week of the event [27]. However, both resident and staff surgeons agree that postoperative feedback is given far less often than needed [28]. Moreover, surgical residents desire better feedback during residency to grow and develop as leaders [29].

Nevertheless, the studies pertinent to this topic differ substantially with respect to methodology, population, context, and objective, which makes it even more difficult to achieve a clear understanding of the issue. Among all knowledge synthesis methodologies, scoping reviews are considered to be the best way to present a broad overview of evidence in heterogeneous scenarios, summarizing and promoting better comprehension of a field [30-32]. In addition, despite the relevance of the theme, in a preliminary search, few scoping reviews were found on this topic. Although all of these scoping reviews focused on surgery residents’ training in some manner [33-35], none focused on their feedback perception during and after their training. Therefore, it is evident that a scoping review on this theme would have an unequivocal contribution to the understanding and enhancement of surgical residents’ learning progress.

### Study Aim

The aim of this scoping review is to identify and map out data from studies that report surgical residents’ perception of the feedback received during their education, analyze these themes, determine knowledge gaps, and disseminate the research findings.

## Methods

### Design

This scoping review will be carried out rigorously and transparently using the first 5 stages of the structure proposed by Arksey and O’Malley’s [36]: (1) identify the research question; (2) identify relevant studies; (3) select studies; (4) map out the data; and (5) collate, summarize, and report the results. As this is an emerging field, more studies on the theme will likely be finalized. Although the 6th stage of Arksey and O’Malley’s [36] structure (consulting) will not be completed in this review, the results of the review can inform this stage in a future study. This structure is congruent with the Joanna Briggs Institute’s (JBI) scoping review methodology [32].

### Research Question

The research question was elaborated according to the objective of the review and through the Population, Concept, Context model, with the population including resident physicians of any surgical specialty under the concept of feedback perception in the context of surgical education. Therefore, the following research question was established: “What is known about surgical residents’ perception of feedback in their training?”

### Inclusion Criteria

The main eligibility criteria to include articles in the study are those including a population comprising resident physicians of any surgical specialty and age group who were attending any year of residency. Studies assessing the target population’s perception of feedback will be included, regardless of the type of feedback given and the way the perceptions were measured. Eligible studies will be those related to the teaching of the population in question, focusing on surgical education in any country. The following types of articles will be included in the review: studies with qualitative and quantitative approaches, primary studies, systematic reviews, meta-analyses and/or meta-syntheses, books, and guidelines published in indexed sources.

### Exclusion Criteria

Any studies not meeting the eligibility criteria, not published in indexed sources; and publications of opinions, consensuses, retractions, editorials, websites, and advertisements published in the media will be excluded from the review.

### Search Strategy

The search strategy will be determined by a librarian who is a specialist in digital search strategies based on the following descriptors: formative feedback, hospital medical staff, teaching, general surgery, and perception, with their corresponding terms in the Portuguese and Spanish languages, encompassing the period from 2017 to the search date. For the combination of descriptors, the Boolean operators “AND,” “OR,” and “NOT” will be considered. The full search strategy that will be used is detailed in Multimedia Appendix 1.

We will consult the databases of journals indexed in Medline, Directory of Open Access Journals, Directory of Open Access Scholarly Resources, Academic Search Premier, BioMed Central Open Access, and Wiley-Blackwell, using the descriptors and/or synonyms according to *Descritores em Ciências da Saúde* (Health Science Descriptors) and Medical Subject Headings for each item of the strategy. These databases were selected because they are comprehensive and have broad coverage of publications in the area of health. The choice of databases will depend on the research question.

### Study/Source of Evidence Selection

Duplicate articles will be excluded manually. For the remaining articles, titles and abstracts will be analyzed by 2 independent reviewers to select those that meet the inclusion criteria. Articles that do not meet the eligibility criteria described above will be excluded. In case of divergence, a third reviewer will be consulted and will give the final opinion about the relevance of the article for answering the main research question. Additional sources can be included in the review after a manual search performed by the reviewers, as long as they meet the eligibility criteria, are important to complete the study, and have not already been identified by the search strategy.

To align the inclusion criteria among the reviewers, the titles and abstracts of 25 random articles will be analyzed by 3 researchers. Disagreements regarding the inclusion or exclusion of the articles will be discussed until a consensus is reached.

The complete texts of the selected articles will be evaluated by the main researcher (CDdSC) based on the inclusion criteria. The reasons for the exclusion of articles that have undergone full-text review will be registered and reported in the scoping review report. Any disagreement that emerges among the researchers at any stage of the selection process will be solved through discussion or the addition of other researchers. A PRISMA-ScR (Preferred Reporting Items for Systematic Reviews and Meta-Analyses Extension for Scoping Reviews) flow diagram [36-39] will be used to present, in full, the results of the process of search and inclusion of studies in the scoping review.

### Data Extraction

After article selection, the main researcher (CDdSC) will create a form for data extraction, which will be filled in after reading of the full text of the article. The extracted data will include specific details about participants, concept, context, and methods of study, as well as key information related to the research question, such as the method and model of feedback delivery, the strategy used, the study population’s perceptions, and impact of these perceptions on surgical resident education, when such information is available.

A draft of the data extraction form is provided in Textbox 1. This form can be modified and revised as the need arises during the process of data extraction from each included source. The modifications will be described in the scoping review. If appropriate, the authors of the articles will be contacted and asked about missing or complementary data when necessary.

**Data extraction instrument developed by the researchers.**

**Publication details**

- Journal
- Year
- Title
- Author
- Country
- Type of study

**Inclusion/exclusion criteria**

- Participants
- Concept
- Context
- Reason for exclusion

**Findings**

- Method of feedback
- Model of feedback
- Resident’s perception
- Impact

### Analysis of the Evidence

The data will be analyzed in light of the study’s objectives. Quantitative and qualitative analyses will be performed through calculation of descriptive statistics (eg, absolute and percentage frequencies) and content analysis, respectively [40]. Basic data coding will be used for the qualitative analyses, if necessary, depending on the findings. The results will be presented graphically and in the form of tables. This information will be enriched by a descriptive text that will clearly show how the results are related to the research question.

## Results

The findings of this scoping review were submitted to an indexed journal in July 2024, currently awaiting reviewer approval. The search was executed on March 15, 2024, and resulted in 588 articles. After the exclusion of the duplicate articles and those that did not meet the eligibility criteria as well as the inclusion of articles through a manual search, 13 articles were included in the review.

## Discussion

### Projected Significance

This protocol was designed according to the first 5 stages of Arksey and O’Malley’s [36] structure and is congruent with the JBI methodology. Conducting a scoping review is the best way to map what is known about surgical residents’ perception of the feedback received during their education, which can further help to analyze related concepts and determine gaps in the published literature about this subject. Studies in this field have largely focused on the quality of the information to be transmitted, how to transmit such information, and what would be the best moment to do so. However, recent studies on the subject have revealed a shift in priority toward understanding the progress achieved in learning and the learner’s engagement in the process enabled by the feedback.

For example, understanding residents’ perceptions of the feedback received according to the method and model used to provide the feedback could help educators reconsider how to effectively reach their educational objectives in surgery education. As part of knowledge translation [41], such understanding could even result in the proposal of modifications to the medical curriculum and its development, which could be the topic of future research.

### Limitations

This protocol has an important limitation. The search strategy was established using terms in Portuguese, Spanish, and English, which surely excludes articles published in different languages and cultures. Moreover, it is known that feedback concepts and practices differ according to the culture and environment [42]. Thus, the findings of this scoping review may not depict the perception of feedback worldwide.

### Conclusions

Feedback is an essential tool for learning and improving performance in any sphere of education. Although its mechanism is not fully known, the delivery of feedback to residents continues to be an important area of study in medical teaching. Despite the relevance of the theme, the methodologies, populations, and contexts of the few studies available that are pertinent to the matter differ markedly from one another, and a scoping review on this topic would unequivocally enhance and organize what is already known. In addition, by focusing on the feedback perception more than the feedback itself, the results of this scoping review will contribute to gaining a better understanding of how to proceed to enhance internal feedback and residents’ learning progress.

## Appendix

Multimedia Appendix 1 Full search strategy.

## Abbreviations

JBI: Joanna Briggs Institute
PRISMA-ScR: Preferred Reporting Items for Systematic Reviews and Meta-Analyses Extension for Scoping Reviews
