# Supplementary material for: Surgical Residents’ Perception of Feedback on Their Education: Protocol for a Scoping Review
Source: JMIR Res Protoc. 2024 Aug 19;13:e56727. doi: 10.2196/56727 (PMC11369536; doi:10.2196/56727)
Supplement: Multimedia Appendix 1 [file resprot_v13i1e56727_app1.docx]

Multimedia appendix 1

Full search strategy.

| Subjects | Subject and synonyms in Portuguese (DeCS^a^) | Subject and synonyms in English (MeSH^b^) | Subject and synonyms in Spanish (DeCS) |
| --- | --- | --- | --- |
| Subject 1 | “*Feedback Formativo*” | “Formative Feedback” OR “Feedback, Formative” OR “Constructive Feedback” OR “Feedback, Constructive” OR “Feedback (Learning)” | *“Retroalimentación Formativa”* |
| Subject 2 | “*Corpo Clínico Hospitalar*” OR “*Chefe de Serviços Médicos Hospitalares*” OR “*Corpo Médico Hospitalar*” OR “*Médico Residente*” OR “*Médicos Recém-Formados*” OR “*Médicos Residentes*” | “Medical Staff, Hospital” OR “Hospital Medical Staff” OR “Hospital Medical Staffs” OR “Staff, Hospital Medical” OR “Staffs, Hospital Medical” OR “Medical Staffs, Hospital” OR “Physicians, Junior” OR “Junior Physician” OR “Junior Physicians” OR “Physician, Junior” OR “Registrars, Hospital” OR “Hospital Registrar” OR “Hospital Registrars” OR “Registrar, Hospital” OR “Attending Physicians, Hospital” OR “Hospital Attending Physician” OR “Hospital Attending Physicians” OR “Attending Physician, Hospital” | *“Cuerpo Médico de Hospitales”* |
| Subject 3 | “*Ensino*” OR “*Atividade de Treinamento*” OR “*Atividades Formativas*” OR “*Atividades de Capacitação*” OR “*Atividades de Formação*” OR “*Atividades de Treinamento*” OR “*Atividades de Treino*” OR “*Capacitação Acadêmica*” OR “*Didática*” OR “*Docência*” OR “*Formação Acadêmica*” OR “*Método de Ensino*” OR “*Métodos Pedagógicos*” OR “*Métodos de Ensino*” OR “*Pedagogia*” OR “*Treinamento Acadêmica*” OR “*Treino Acadêmico*” OR “*Técnica de Treinamento*” OR “*Técnicas Educacionais*” OR “*Técnicas Educativas*” OR “*Técnicas de Ensino*” OR “*Técnicas de Formação*” OR “*Técnicas de Treinamento*” OR “*Técnicas de Treino*” | “Teaching” OR “Training Techniques” OR “Training Technique” OR “Technique, Training” OR “Techniques, Training” OR “Training Technics” OR “Technic, Training” OR “Technics, Training” OR “Training Technic” OR “Pedagogy” OR “Pedagogies” OR “Teaching Methods” OR “Teaching Method” OR “Method, Teaching” OR “Methods, Teaching” OR “Academic Training” OR “Training, Academic” OR “Training Activities” OR “Training Activity” OR “Activities, Training” OR “Activity, Training” OR “Techniques, Educational” OR “Educational Techniques” OR “Educational Technique” OR “Technique, Educational” OR “Educational Technics” OR “Educational Technic” OR “Technic, Educational” OR “Technics, Educational” | *“Enseñanza”* |
| Subject 4 | “*Cirurgia Geral*” | “General Surgery” OR “Surgery, General” OR “Surgery” | *“Cirugía General”* |
| Subject 5 | “*Percepção*” | “Perception” | *“Percepción”* |

^a^DECS: *Descritores em Ciências da Saúde*

^b^MeSH: Medical Subject Headings
